# Higher prevalence of obesity among children with functional abdominal pain disorders

**DOI:** 10.1186/s12887-020-02106-9

**Published:** 2020-05-06

**Authors:** Tut Galai, Hadar Moran-Lev, Shlomi Cohen, Amir Ben-Tov, Dina Levy, Yael Weintraub, Achiya Amir, Or Segev, Anat Yerushalmy-Feler

**Affiliations:** Pediatric Gastroenterology Unit, “Dana-Dwek” Children’s Hospital, Tel Aviv Sourasky Medical Center and the Sackler Faculty of Medicine, Tel Aviv University, 6 Weizmann Street, 6423906 Tel Aviv, Israel

**Keywords:** Functional gastrointestinal disorders, Children, Irritable bowel syndrome, Overweight, Obesity

## Abstract

**Background:**

Functional abdominal pain (FAP) disorders are one of the most common gastrointestinal disorders in children. We aimed to define the association between obesity and functional abdominal pain (FAP) disorders and to assess differences between overweight/obese children and normal weight children with FAP disorders.

**Methods:**

We conducted a retrospective study of children (2–18 years old) with a clinical diagnosis of FAP who were followed-up in our pediatric gastroenterology unit between 1/2016–10/2018. FAP disorders were defined according to the ROME IV criteria. Body mass index (BMI) percentiles were defined by CDC standards. Patients with BMIs ≥85th percentile were designated as being overweight/obese. A population control group was obtained from the 2015–2016 Israel national health survey.

**Results:**

Data from 173 children with FAP disorders (median age 11.5 years, 114 females) were included. Seventy-one children (41%) were classified as having functional abdominal pain-NOS, 67 (38.7%) as having irritable bowel syndrome (IBS), and 35 (20.2%) has having functional dyspepsia. Fifty-three children (30.6%) were classified as being overweight/obese.

Adolescents with FAP disorders had a significantly higher prevalence of overweight/obesity compared to controls (39.5% vs. 30%, respectively, *p* = 0.04). Children with FAP and overweight were older [12.4 (range 9.8–15.3) vs. 10.8 (7.4–14.1) years, *p* = 0.04] and had more hospitalizations due to FAP (20.8% vs. 7.6%, *p* = 0.01) compared to Children with FAP and normal weight.

**Conclusions:**

Adolescents with FAP had higher prevalence of overweight/obesity compared to controls. Future studies are warranted to raise awareness of weight issues in FAP and determine the effect of weight loss on FAP.

## Background

Functional gastrointestinal disorders (FGID) are very common clinical conditions encountered in day-to-day clinical pediatric gastroenterology practice [1]. Increased knowledge of the pathophysiology of FGID in the last decade has led to the design of a biopsychosocial model in which genetic, physiological, and psychological factors interact [2]. The diagnosis of FGID is clinical and based on the Rome diagnostic criteria, which have evolved during the last decades to form the newest set, the Rome IV criteria which was issued in 2016 [3]. Several epidemiological studies using the Rome III and Rome IV criteria showed that FGID related to abdominal pain are highly prevalent [4, 5]. According to the Rome IV criteria, the term “abdominal pain related functional gastrointestinal disorders” has been changed to “functional abdominal pain disorders” and it includes functional dyspepsia (FD), irritable bowel syndrome (IBS), abdominal migraine, and functional abdominal pain not otherwise specified (functional abdominal pain NOS) [3].

Childhood obesity is a serious and urgent public health problem that is associated with physical and psychological comorbidities [6]. The bidirectional association between obesity and FGID has been described in previous studies, however, some of them are limited by a lack of control groups or outdated diagnostic criteria [7–9], and none of them was based on ROME IV criteria. The primary aim of this study was to define the association between obesity and functional abdominal pain (FAP) disorders in children according to the Rome IV criteria compared with a population control, and to describe and compare clinical characteristics of children with overweight/obesity with those of healthy weight and FAP disorders.

## Methods

All study procedures were approved by the institutional review board of the Tel Aviv Medical Center (Helsinki Committee), and parental informed consent was obtained for all participants.

### Study design and participants

We conducted a retrospective study of 2- to 18-year-old children with a clinical diagnosis of FAP disorders that were followed-up in the Pediatric Gastroenterology Unit at the “Dana-Dwek” Children’s Hospital, Tel Aviv, Israel, between January 2016 and October 2018. The Pediatric Gastroenterology unit is an outpatient clinic at our hospital, which is a tertiary referral center for pediatric gastroenterology. The patients were identified through the local electronic health records database. After the patients with clinical diagnosis of FAP were identified from the medical records, we performed an objective evaluation of the compatibility of their diagnosis with the ROME IV criteria [3]. Patients that did not fulfill these criteria were excluded from the study, as were patients with missing anthropometric data. Body mass index (BMI) percentiles were defined by the CDC standards (https://www.cdc.gov/growthcharts/clinical_charts.htm). Patients with BMIs ≥85th percentile were designated as being overweight or obese.

A population control group was obtained from the 2015–2016 Israel national health survey (https://www.health.gov.il/UnitsOffice/ICDC/mabat/Pages/default.aspx). The Israeli health survey is a national survey, conducted every 3–4 years by the ministry of health of Israel, that aims to investigate health and nutrition status among the Israeli population by a representative sample of children and adults. The data in the survey is presented according to age groups (2–11 and 12–18 years of age) and gender.

### Data collection

We retrospectively reviewed the patients’ records and collected demographic data consisting of age, gender, location and duration of symptoms (abdominal pain, vomiting, diarrhea, heartburn) as well as anthropometric data (weight, height, BMI). We also reviewed imaging and endoscopies studies, hospitalizations, dietitian consultations, nutrition recommendations, and follow-up durations.

### Data statistical analysis

Categorical variables were reported as frequency and percentage. Continuous variables were evaluated for normal distribution using a histogram and Kolmogorov–Smirnov test. Since none of the continuous variables were normally distributed, they were reported as median and interquartile range (IQR). Overweight proportion was compared to the published data using the one-sample binomial test. Characteristics of overweight and non-overweight children were compared using the Chi-square test or Fisher’s exact test for the categorical variables, and the Mann-Whitney test was used for the continuous and ordinal variables. All statistical tests were two-sided, and *p* < 0.05 was considered statistically significant. SPSS was used for all statistical analyses (IBM SPSS Statistics, version 25, IBM Corp., Armonk, NY, USA).

## Results

### Demographic and clinical data

A total of 274 children were identified as having the diagnosis of FAP. Forty-eight children who did not meet the ROME IV criteria and 53 children with missing data were excluded, leaving a total of 173 children for final analysis. Of these children, 114 (65.8%) were females, and the group’s median age was 11.5 (IQR 7.9–14.5) years, nearly all of them were from Israeli Jewish origin (170 patients, 98.3%). The functional disorders were functional abdominal pain NOS (*n* = 71, 41%), IBS (*n* = 67, 38.8%), and FD (*n* = 35, 20.2%). No abdominal migraine cases were identified. The median duration of the abdominal pain was 8 months (IQR 4–20), the median BMI percentile was 64 (IQR 26.7–86.5), and the median follow-up duration was 2.1 months (IQR 0–6). The demographic and clinical data are presented in Table 1.

**Table 1:** Demographic and clinical data

	Study cohort *N* = 173	Normal or underweight *N* = 120 (69.3%)	Overweight or obese *N* = 53 (30.7%)	P
Age (years)	11.5 (7.9–14.5)	10.8 (7.4–14.1)	12.4 (9.8–15.3)	0.04
Females	114 (65.8%)	76 (63.3%)	38 (71.7%)	0.29
FAP disorders:				0.07
Functional abdominal pain NOS	71 (41%)	55 (45.8%)	16 (30.2%)	
Irritable bowel syndrome	67 (38.8%)	45 (37.5%)	22 (41.5%)	
Functional dyspepsia	35 (20.2%)	20 (16.7%)	15 (28.3%)	
Hospitalizations	20 (11.6%)	9 (7.5%)	11 (20.8%)	0.01
Proton pump inhibitors treatment	38 (22%)	24 (20%)	14 (26.4%)	0.05
Dietitian consultations	46 (26.6%)	23 (19.2%)	23 (43.4%)	0.001

Sixty children (36.4%) underwent gastroscopy, 13 (7.5%) underwent colonoscopy, and 132 (76.3%) underwent imaging studies, of whom 115 (66.5%) had an abdominal ultrasound and 17 (9.8%) had an abdominal computerized tomography or a magnetic resonance enterography. Ninety-nine children (57.2%) received medications: 50 (28.9%) were treated with stool softeners, 38 (22%) with proton pump inhibitors (PPI), and 11 (6.4%) with probiotics. No gender difference was noticed in medication use. However, significantly more children with FD were treated with PPI compared to children with IBS and functional abdominal pain NOS (57.1, 13.4 and 12.7%, respectively, *P* < 0.001). Twenty children (11.5%) were hospitalized due to FAP. In addition, 105 children (60.6%) were given general balanced dietary recommendations from the physician and 46 (26.5%) children received formal dietitian consultations. Specific dietary recommendations were given as follows: high fiber (*n* = 38, 22%), reduced simple carbohydrates (*n* = 24, 13.9%) or lactose free (*n* = 19, 11%).

### Overweight/obese vs. normal weight in FAP group

Of all 173 children, 53 children (30.6%) were accounted as overweight/obese. Among these 53 children, 20 were truly obese (37.7%), 15 (28.3%) boys and 38 (71.7%) girls. The distribution of FAP disorders among the children with overweight/obesity was as follow; 22 (41.5%) with IBS, 16 (30.2%) with functional abdominal pain-NOS and 15 (28.3%) with FD (Table 1). No significant relation was found between obesity and FAP subgroups. Children with FAP and overweight/obesity disorders were older (12.4 (9.8–15.3) vs. 10.8 (7.4–14.1), *p* = 0.04), had more hospitalizations due to FAP (20.8% vs. 7.5% *p* = 0.01), were treated more with PPI (26.4% vs. 20%, *p* = 0.05) and had more dietitian consultations (43.4% vs. 19.2%, *p* = 0.001) compared with children with FAP and normal weight (Table 1). There was no significant difference in gender, endoscopies and imaging studies performance, follow up duration and nutrition recommendations between the groups.

### The FAP group vs. the controls

The 6432 controls were divided into two age groups, one 2–11 years of age and one 12–18 years of age. The younger age group included 1792 children, of whom 919 (51.3%) were boys and 873 (48.7%) were girls. There were 299 children (16.7%) in the young age group who were designated as having overweight/obesity. The older age group of 4640 children included 2223 (47.9%) boys and 2417 (52%) girls, among whom 1433 (30.8%) were designated as having overweight/obesity (Table 2). The 2- to 11-year-old girls with FAP had a significantly higher prevalence of overweight/obesity compared with the controls in that age range (70.6% vs. 48.7%, *p* = 0.04) (Table 2). In addition, the 12- to 18-year old children with FAP had a significantly higher prevalence of overweight/obesity compared to controls (39.5% vs. 30.8%, respectively, *p* = 0.04), and specifically in girls (42.9% vs. 29.2%, *p* = 0.01) in that age range. There was no significant difference between prevalence of overweight/obesity of boys with FAP in any age group compared to controls.

**Table 2:** Overweight Patients in the FAP and Control Groups by Age and Gender

Children 12–18 years old	FAP group *N* = 81	Control *N* = 4640	P
Total overweight/obese	32 (39.5%)	1433 (30.8%)	0.04
Males	32 (39.5%)	2223 (47.9%)	0.44
Overweight/obese males	11 (34.4%)	726 (32.6%)	
Females	49 (60.4%)	2417 (52%)	0.01
Overweight/obese females	21 (42.9%)	707 (29.2%)	
**Children 2–11 years old**	**FAP group** ***N*** **= 92**	**Control*N*** **= 1792**	**P**
Total overweight/obese	21 (22.8%)	299 (16.7%)	0.07
Males	27 (29.3%)	919 (51.3%)	0.5
Overweight/obese males	4 (14.8%)	148 (16.1%)	
Females	65 (70.6%)	873 (48.7%)	0.04
Overweight/obese females	17 (26.2%)	151 (17.3%)	

## Discussion

The association between obesity and FAP according to the updated ROME IV criteria had not been investigated before. In the current study, our analysis of that association revealed a higher prevalence of overweight/obesity among adolescents with FAP disorders compared to an age- and sex-matched control population. Our results are in line with studies that had demonstrated a correlation between body weight and FGID. Teitelbaum et al. [8] showed a greater percentage of children with obesity and constipation, gastroesophageal reflux disease, IBS, encopresis and FAP compared with children with healthy weight. In their study, functional disorders were assigned based on ROME II criteria without specification of subtypes of FAP. From another point of view, Bonilla et al. [9] described a cohort from 2007 to 2008 with a prevalence of 20.2% obesity in patients with FGID, however, no comparison to healthy control group was performed. They showed that obesity was associated with poor outcome and disability at long term follow up. In our study the prevalence of overweight/obesity was higher, probably attributed to the combination of children with overweight and obesity as one group or to the increase in prevalence of obesity in the western world as part of the obesity epidemic. Other studies demonstrated higher percentages of recurrent abdominal pain [10] and FGID [7] in children with obesity. However, Malaty et al. [10] used a non-validated questionnaire and the latter study was based on ROME III criteria for diagnosis of functional disorders.

Several factors may explain the association between obesity and FAP disorders. Dietary habits are a major factor in obesity development and previous studies showed the association between increased consumption of carbohydrates and high body weight [11–13]. Carbohydrate malabsorption may cause gastrointestinal symptoms via the physiologic effects of both increased osmotic activity and increased gas production from bacterial fermentation [14]. Moreover, there is some evidence that a low-FODMAP diet is effective in reducing IBS symptoms [15, 16]. Recently, Schnabel et al. showed an association between ultra-processed food (UPF) consumption and functional gastrointestinal disorders. In this large French cohort, an increase in UPF, which is characterized by high density of saturated fatty acids, sugar, sodium and low content of protective nutrients such as fibers, was associated with a higher risk of IBS. They also found that UPF consumption was associated with higher BMI [17].. In addition to dietary habits, sedentary lifestyle and lack of physical activity could be related to both obesity and functional pain [18].

A potential association between obesity, FGID and gastrointestinal motility disorders has also been described. Delayed gastric emptying and impaired antral motility were found in children with RAP, FAP, IBS or functional dyspepsia [19, 20]. Several studies have shown delayed gastric emptying and gastric and gallbladder dysmotility in the individuals with obesity [21, 22]. This may be attributed to increased gastric distention in the obese causing poor fundal and antral tone [23], altered sensitivity of mechanoreceptors in the stomach musculature [24] and abnormal perception of satiety signals [25].

Another association between obesity and FGID is the gut microbiota. Increased risk of small intestinal bacterial overgrowth [26] and different gut microbiota composition [27] in obesity has been reported which might contribute to gastrointestinal dysmotility, excessive fermentation, altered visceral perception and gut permeability with their metabolites leading to pain-predominant FGID [28, 29]. Finally, obesity and FGID share common psychological comorbidities, such as stress, depression, and anxiety, which can contribute to each other’s development and aggravate each other [30–32].

The higher prevalence of overweight/obesity in females with FAP compared to controls may be attributed to several factors. Females are more prone to several gastrointestinal motility disorders, such as delayed gastric emptying, compared to males [33, 34]. It has been suggested that this difference may be caused by female ovarian hormones. Gender differences in intestinal microbiome in addition to microbiome difference between children with overweight/obesity and children with healthy weight [35] may serve as an alternative explanation.

Our study has demonstrated a few significant differences between children with FAP having overweight/obesity compared to normal weight children. We found that children with overweight/obesity had more hospitalizations attributed to their abdominal pain compared with children with healthy weight, as described in other studies which suggested that pediatric obesity contributes significantly to increased health care utilization in children [36, 37]. A possible explanation to increased health care utilization in children with obesity is comorbidities of obesity [36]. In addition, children with obesity might be more symptomatic compared to children with normal weight [38]. We also found that obese children with FAP are treated more frequently with PPI than non-obese children with FAP. Parkman et al. [34] reported that patients with obesity and idiopathic gastroparesis tend to be more symptomatic compared to patients with healthy weight. Whether this is true to patients with FAP is to be elucidated. Although no relation between FAP subgroups and obesity was established in this study, children with FD were treated more frequently with PPI, in line with the ROME IV recommendations [3], compared to other FAP subgroups. Generally, overuse of PPI has been increasing in the last decade in hospitalized and ambulatory patients and their prescription continues to grow in all western countries [39].

The clinical implications of the findings of our current study relate to the management of children with overweight/obesity and FGID. Although this study could not indicate causality between obesity and FAP, the findings support a relation between these conditions. The treatment protocol of children with obesity and FAP should also focus on guidance for more thorough nutritional assessment targeted towards weight reduction together with other lifestyle changes (e.g., increase in physical activities) that may improve symptoms and prevent or at least minimize the need for medications and hospitalizations.

To the best of our knowledge, this is the first study to show an association between obesity and FAP diagnosed in children according to the updated ROME IV criteria for diagnosis of FAP, which was made by a pediatric gastroenterologist in a clinic setting and not by collecting information from a self-administered questionnaire. This study is limited by its retrospective nature, missing more precise data on skinfold measurements, body composition, and other parameters. In addition, our control group might include children with FAP disorders, although we believe that due to the high number of children included, the percentage of children with FAP disorders would be similar to the general population. Lastly, a part of children with diagnosis of FAP in the medical records did not fulfil the ROME IV criteria and therefore were excluded. In real life, some degree of incompatibility between clinical diagnosis and formal criteria may be anticipated. Another explanation is the difference between ROME IV and ROME III criteria, that was one of the rationales to conduct the current study.

## Conclusions

Adolescents with FAP disorders have a higher prevalence of overweight/obesity compared with controls in the general population. Children with FAP and overweight/obesity have more hospitalizations and medical treatment with PPI compared to children with FAP disorders who are of normal weight. Heightened awareness of these findings might improve treatment planning and management to meet the needs of children with overweight/obesity and FAP. Further studies should focus on the interrelationship between FGID and obesity and specifically on the pathophysiologic mechanisms between these conditions, and also to investigate the effect of weight reduction on FAP symptoms.

## Data Availability

∙The datasets used and/or analysed during the current study are available from the corresponding author on reasonable request.

## Abbreviations

FGID: Functional gastrointestinal disorders
FD: Functional dyspepsia
IBS: Irritable bowel syndrome
NOS: Not otherwise specified
FAP: Functional abdominal pain
BMI: Body mass index
IQR: Interquartile range
PPI: Proton pump inhibitors

## References

[1] Rouster AS, Karpinski AC, Silver D (2016). Functional gastrointestinal disorders dominate pediatric gastroenterology outpatient practice. J Pediatr Gastroenterol Nutr.

[2] Korterink J, Devanarayana NM, Rajindrajith S (2015). Childhood functional abdominal pain: mechanisms and management. Nat Rev Gastroenterol Hepatol.

[3] Rasquin A, Di Lorenzo C, Forbes D (2016). Childhood functional gastrointestinal disorders: child/adolescent. Gastroenterology.

[4] Devanarayana NM, Rajindrajith S, Perera MS (2014). Association between functional gastrointestinal diseases and exposure to abuse in teenagers. J Trop Pediatr.

[5] Saps M, Velasco-Benitez CA, Langshaw AH (2018). Prevalence of functional gastrointestinal disorders in children and adolescents: comparison between Rome III and Rome IV criteria. J Pediatr.

[6] Sanders RH, Han A, Baker JS (2015). Childhood obesity and its physical and psychological co-morbidities: a systematic review of Australian children and adolescents. Eur J Pediatr.

[7] Tambucci R, Quitadamo P, Ambrosi M (2019). Association between obesity/overweight and functional gastrointestinal disorders in children. J Pediatr Gastroenterol Nutr.

[8] Teitelbaum JE, Sinha P, Micale M (2009). Obesity is related to multiple functional abdominal diseases. J Pediatr.

[9] Bonilla S, Wang D, Saps M (2011). Obesity predicts persistence of pain in children with functional gastrointestinal disorders. Int J Obes.

[10] Malaty HM, Abudayyeh S, Fraley K (2007). Recurrent abdominal pain in school children: effect of obesity and diet. Acta Paediatr.

[11] Emmett PM, Jones LR (2015). Diet, growth, and obesity development throughout childhood in the Avon longitudinal study of parents and children. Nutr Rev.

[12] Te Morenga L, Mallard S, Mann J (2012). Dietary sugars and body weight: systematic review and meta-analyses of randomised controlled trials and cohort studies. BMJ.

[13] Dello Russo M, Ahrens W, De Henauw S (2018). IDEFICS Consortium. The Impact of Adding Sugars to Milk and Fruit on Adiposity and Diet Quality in Children: A Cross-Sectional and Longitudinal Analysis of the Identification and Prevention of Dietary- and Lifestyle-Induced Health Effects in Children and Infants (IDEFICS) Study. Nutrients.

[14] Chumpitazi BP, Shulman RJ (2016). Dietary carbohydrates and childhood functional abdominal pain. Ann Nutr Metab.

[15] Shepherd SJ, Lomer MC, Gibson PR (2013). Short-chain carbohydrates and functional gastrointestinal disorders. Am J Gastroenterol.

[16] Staudacher HM, Whelan K, Irving PM (2011). Comparison of symptom response following advice for a diet low in fermentable carbohydrates (FODMAPs) versus standard dietary advice in patients with irritable bowel syndrome. J Hum Nutr Diet.

[17] Schnabel L, Buscail C, Sabate JM (2013). Association between ultra-processed food consumption and functional gastrointestinal disorders: results from the French NutriNet-Santé cohort. JPGN.

[18] Rabbitts JA, Holley AL, Karlson CW, Palermo TM (2014). Bidirectional associations between pain and physical activity in adolescents. Clin J Pain.

[19] Devanarayana NM, Rajindrajith S, Bandara C (2013). Ultrasonographic assessment of liquid gastric emptying and antral motility according to the subtypes of irritable bowel syndrome in children. J Pediatr Gastroenterol Nutr.

[20] Devanarayana NM, Rajindrajith S, Perera MS (2013). Gastric emptying and antral motility parameters in children with functional dyspepsia: association with symptom severity. J Gastroenterol Hepatol.

[21] Di Ciaula A, Wang DQ, Portincasa P (2012). Gallbladder and gastric motility in obese newborns, pre-adolescents and adults. J Gastroenterol Hepatol.

[22] Jackson SJ, Leahy FE, McGowan AA (2004). Delayed gastric emptying in the obese: an assessment using the non-invasive (13) C-octanoic acid breath test. Diabetes Obes Metab.

[23] Horowitz M, Collins PJ, Cook DJ (1983). Abnormalities of gastric emptying in obese patients. Int J Obes.

[24] Maddox A, Horowitz M, Wishart J (1989). Gastric and oesophageal emptying in obesity. Scand J Gastroenterol.

[25] Horowitz M, Collins PJ, Shearman DJ (1986). Effect of increasing the caloric/osmotic content of the liquid component of a mixed solid and liquid meal on gastric emptying in obese subjects. Hum Nutr Clin Nutr.

[26] Roland BC, Lee D, Miller LS (2018). Obesity increases the risk of small intestinal bacterial overgrowth (SIBO). Neurogastroenterology Motility.

[27] Hou YP, He QQ, Ouyang HM, et al. Human gut microbiota associated with obesity in Chinese children and adolescents. Biomed Res Int. 2017;758598:1-8. 10.1155/2017/7585989 PMC5682041 29214176

[28] Rhee SH, Pothoulakis C, Mayer EA (2009). Principles and clinical implications of the brain–gut–enteric microbiota axis. Nat Rev Gastroenterol Hepatol.

[29] Ohman L, Simren M (2013). Intestinal microbiota and its role in irritable bowel syndrome (IBS). Curr Gastroenterol Rep.

[30] Topçu S, Orhon FŞ, Tayfun M (2016). Anxiety, depression and self-esteem levels in obese children: a case-control study. J Pediatr Endocrinol Metab.

[31] Shin NY, Shin MS (2008). Body dissatisfaction, self-esteem, and depression in obese Korean children. J Pediatr.

[32] Campo JV, Gilchrist RH (2009). Psychiatric comorbidity and functional abdominal pain. Pediatr Ann.

[33] Zia JK, Heitkemper MM (2016). Upper gastrointestinal tract motility disorders in women, Gastroparesis, and Gastroesophageal reflux disease. Gastroenterol Clin N Am.

[34] Parkman HP, Yates K, Hasler WL (2011). Clinical features of idiopathic gastroparesis vary with sex, body mass, symptom onset, delay in gastric emptying, and gastroparesis severity. Gastroenterology.

[35] Gao X, Jia R, Xie L (2018). A study of the correlation between obesity and intestinal flora in school-age children. Sci Rep.

[36] Hampl SE, Carroll CA, Simon SD (2007). Resource utilization and expenditures for overweight and obese children. Arch Pediatr Adolesc Med.

[37] Trasande L, Chatterjee S (2009). The impact of obesity on health service utilization and costs in childhood. Obesity..

[38] Grout RW, Thompson-Fleming R, Carroll AE (2018). Prevalence of pain reports in pediatric primary care and association with demographics, body mass index, and exam findings: a cross-sectional study. BMC Pediatr.

[39] Lanas A (2016). We are using too many PPIs, and we need to stop: a European perspective. Am J Gastroenterol.

